# RBM24 exacerbates bladder cancer progression by forming a Runx1t1/TCF4/miR-625-5p feedback loop

**DOI:** 10.1038/s12276-021-00623-w

**Published:** 2021-05-21

**Authors:** Yue-Wei Yin, Kai-Long Liu, Bao-Sai Lu, Wei Li, Ya-Lin Niu, Chen-Ming Zhao, Zhan Yang, Ping-Ying Guo, Jin-Chun Qi

**Affiliations:** 1 Department of Urology, The Second Hospital of Hebei Medical University, Shijiazhuang, 050000 P.R. China; 2 Hebei Institute of Urology, Shijiazhuang, 050000 P.R. China

**Keywords:** Prostate cancer, Cell growth

## Abstract

RNA–binding motif protein 24 (RBM24) acts as a multifunctional determinant of cell fate, proliferation, apoptosis, and differentiation during development by regulating premRNA splicing and mRNA stability. It is also implicated in carcinogenesis, but the functions of RBM24 in bladder cancer (BC) remain unclear. In the present study, we revealed that RBM24 was upregulated in BC tissues. Importantly, we found that a higher level of RBM24 was correlated with poor prognosis in BC patients. Overexpression of RBM24 promoted BC cell proliferation, while depletion of RBM24 inhibited BC cell proliferation in vivo and in vitro. Mechanistically, RBM24 positively regulated Runx1t1 expression in BC cells by binding to and enhancing Runx1t1 mRNA stability. Furthermore, Runx1t1 in turn promoted RBM24 expression by interacting with the transcription factor TCF4 and suppressing the transcription of miR-625-5p, which directly targets RBM24 and suppresses RBM24 expression. RBM24-regulated BC cell proliferation was moderated via the Runx1t1/TCF4/miR-625-5p feedback loop. These results indicate that the RBM24/Runx1t1/TCF4/miR-625-5p positive feedback loop participates in BC progression. Disruption of this pathway may be a potential therapeutic strategy for BC treatment.

## Bladder cancer: Tracking down the molecular mechanisms

A protein called RBM24 promotes progression of bladder cancer (BC) by forming a positive feedback loop with a specific transcription factor, driving cancer cell proliferation. Survival rates for BC are low, and the current imperfect understanding of the underlying mechanisms makes it difficult to treat. Ping-Ying Guo at Hebei Medical University in Shijiazhuang, China, and co-workers investigated the role of RBM24, known to be involved in other cancers, and found increased levels in BC tissues. Higher levels were associated with a poor prognosis. Further investigation revealed that RBM24 boosts levels of the transcription factor, which suppresses a molecule that in turn suppresses RBM24, forming a positive feedback loop promoting BC cell proliferation. Interrupting the feedback loop decreased tumor size in a mouse model. These results may help identify better treatments for BC.

## Introduction

Bladder cancer (BC) is one of the most common malignancies in the urinary system, with an estimated 450,000 new cases per year worldwide^1^. Among these cases, approximately 70% of new cases are nonmuscle-invasive BC (NMIBC), while 30% are muscle-invasive BC (MIBC)^2^. Surgery, chemotherapy, and radiation therapy are the main treatments for BC patients^3^. However, the 5-year survival rate of high-risk patients is still very low, and the current main treatment methods cannot prevent the recurrence or progression of these BC patients^4^. One important reason is the poor understanding of the mechanisms underlying BC development and progression. Therefore, it is necessary to better understand the molecular basis of BC and explore innovative therapeutic strategies.

RNA-binding motif protein 24 (RBM24) is a multifunctional protein involved in the regulation of premRNA splicing, mRNA stability, and translation, and through these functions, it acts as a critical determinant of cell fate and differentiation^5^. It was once believed that RBM24 is preferentially expressed in cardiac and skeletal muscle tissues and primarily serves to regulate embryonic heart development^6,7^. However, recent studies have demonstrated that RBM24 also regulates cancer progression^8^. Hua et al. found that RBM24 inhibited the progression of nasopharyngeal carcinoma by upregulating miR-25, which in turn downregulated MALAT1^9^. Using immunoprecipitation coupled to reverse transcription and microarray analysis (RIP-ChIP), Yu et al. demonstrated that RBM24 is a multitasking RNA-binding protein (RBP) capable of regulating the stability and expression of multiple bound targets^10^. RBPs may function as suppressors or facilitators of disease depending on the specific upstream regulators and downstream effectors (targets)^3^. However, the role of RBM24 in BC is still unclear.

Runt-related transcription factor 1 (Runx1t1) is a member of the eight–twenty–one (ETO) family of proteins^11^. Runx1t1 was first identified through its involvement in a t(8;21) translocation associated with acute myeloid leukemia (AML)^12^. Subsequent studies reported that Runx1t1 acts as a transcriptional corepressor by interacting with DNA-bound transcription factors and recruiting other proteins to facilitate transcriptional repression^13^. A recent study found upregulated expression of Runx1t1 in cord blood-derived endothelial colony-forming cells^14^. In accord with the function of Runx1t1 in vascular endothelial development, a Runx1t1-deficient mouse showed reduced angiogenesis^15^. However, Runx1t1 was also reported to suppress colorectal cancer by regulating cell proliferation and chemotherapeutic drug resistance^16^. Runx1t1 may upregulate the levels of the cell cycle genes Cdk4 and Cdk6 by recruiting a histone deacetylase (HDAC)-containing nuclear corepressor complex^17,18^. However, the role of Runx1t1 in BC remains unclear.

In the present study, we found that higher RBM24 and Runx1t1 levels in BC tissue were correlated with poor patient survival in BC patients. Moreover, overexpression of RBM24 upregulated Runx1t1 by stabilizing Runx1t1 mRNA and concomitantly accelerated BC proliferation. We identified a protein–protein interaction between Runx1t1 and TCF4 that suppressed miR-625-5p at the transcriptional level, which acted as a negative regulator of RBM24 by directly targeting RBM24. Taken together, our results indicate that RBM24, Runx1t1, TCF4, and miR-625-5p form a positive feedback loop that can drive the proliferation of BC cells. The RBM24/Runx1t1/TCF4/miR-625-5p pathway may be a potential therapeutic target for BC treatment.

## Materials and methods

### Clinical samples

Human primary BC tissues and the corresponding normal bladder tissues were collected from BC patients who were admitted to the Department of Urology of the Second Hospital of Hebei Medical University from July 2015 to June 2019. All BC patients were histopathologically and clinically diagnosed and were treated with radical cystectomy. The study protocol was approved by the Ethics Committee of Second Hospital of Hebei Medical University, and written consent was obtained from each patient.

### Cell lines and transfection

The normal uroepithelial cell SV-HUC-1 was purchased from the Cell Bank of the Chinese Academy of Sciences, and bladder cancer cell lines UM-UC-3, 253 J, T24, and J82 were purchased from ATCC (Rockville, Maryland). The 293 A cell line was collected in our lab. All cells were cultured in DMEM supplemented with 10% fetal bovine serum (FBS) and 1% penicillin/streptomycin. Cells were grown in a humidified atmosphere of 95% air and 5% CO_2_. Transfection was performed by using Lipofectamine 2000 (Invitrogen) according to the manufacturer’s protocols. The miR-625-5p, miR-149-3p, and miR-449a mimic, mimic NC, miR-625-5p inhibitor, inhibitor NC, shRBM24, shRunx1t1, shTCF4, and negative controls were purchased from GenePharma Co., Ltd. (Shanghai, China). The oeRBM24 and oeRunx1t1 overexpression plasmids were obtained from GENEWIZ Company (Suzhou, China).

### RNA extraction and real-time quantitative PCR

Clinical tumor tissues and xenograft tissues were lysed using QIAzol Lysis Reagent (79306). Total RNA was isolated with a miRNeasy Mini Kit (217004; Qiagen) according to the manufacturer’s protocols. A NanoDrop 2000 was used to determine RNA quality. For miRNA, the miScript II RT Kit (218161) and miScript SYBR Green PCR Kit (Catalog No. 218073) were used to perform reverse transcription and quantitative real-time (qRT)-PCR according to the manufacturer’s protocols with primers listed in Table 1. For mRNA, cDNA was synthesized using an M-MLV First Strand Kit (Life Technologies) with random hexamer primers. mRNAs were subjected to qRT-PCR using the Platinum SYBR Green qPCR SuperMix UDG Kit (Invitrogen) and the ABI 7500 FAST System (Life Technologies) with primers listed in Table 1. The relative transcript expression levels were normalized to GAPDH and calculated using the 2^−ΔΔCt^ formula as previously described^19^.

**Table 1:** Oligos used in this study.

Oligo name	Sequence (5′–3′)	Purpose	Amplicon (bp)
RBM24-F	GCTGGATGCCGGTTGTTAAG	RT-qPCR for RBM24 mRNA.	357
RBM24-R	GCACAAAAGCCTGCGGATAG		
TCF4-F	AGCAGAGTCTCCTTGGAGGT	RT-qPCR for TCF4 mRNA.	206
TCF4-R	AGTGCTTGCTGATGGAGCAT		
RUNX1T1-F	TCACACACAATGTGCCATCCT	RT-PCR for RUNX1T1 mRNA.	273
RUNX1T1-R	TCGGTGAGTCCTGTCTGGAT		
GAPDH-F	AAGGTGAAGGTCGGAGTCAAC	RT-qPCR for GAPDH	102
GAPDH-R	GGGGTCATTGATGGCAACAATA		
miR-29a-F	GGACTGATTTCTTTTGGTGTTCAG	RT-qPCR for miR-29a	N/A
miR-138-3p-F	GCTACTTCACAACACCAGGGC	RT-qPCR for miR-138-3p	N/A
miR-149-3p-F	AGGGAGGGACGGGGGCTG	RT-qPCR for miR-149-3p	N/A
miR-216a-5p-F	GGTAATCTCAGCTGGCAACTGTG	RT-qPCR for miR-216a-5	N/A
miR-222-5p-F	GGCCTCAGTAGCCAGTGTAGATC	RT-qPCR for miR-222-5p	N/A
miR-625-5p-F	GGCAGGGGGAAAGTTCTATAGTCC	RT-qPCR for miR-625-5p	N/A
miR-449a-F	GGTGGCAGTGTATTGTTAGCTGG	RT-qPCR for miR-449a	N/A
miR-578-F	GGCCTTCTTGTGCTCTAGGATTG	RT-qPCR for miR-578	N/A
miR-1273-F	CGGGCGACAAAGCAAGACTC	RT-qPCR for miR-1273	N/A
RNU6-1(U6)-F	GTGCTCGCTTCGGCAGCACATATAC	RT-qPCR for the internal control U6	106
RNU6-1(U6)-R	AAAATATGGAACGCTTCACGAATTTGC		
miR-625-ChIP-1-F	TGGCTCCGCCCCCTTTCAG	ChIP-PCR for miR-625-5p promoter	176
miR-625-ChIP-1-R	GACTGCTGAGCCTGCCACTCC		
miR-625-ChIP-2-F	GAAGTGGCAGCGGGAACAGG	ChIP-PCR for miR-625-5p promoter	225
miR-625-ChIP-2-R	CCAGGAGCAGGCAGCAGCC		
miR-625-ChIP-3-F	CCCAGGAGCCTGTCTGCTTCC	ChIP-PCR for miR-625-5p promoter	167
miR-625-ChIP-3-R	GGATATCATCACAGCCCAACAGG		
RBM24-3′UTR-F	CTCGAGACCAGCCATCTGATCAAAGTTG	PCR amplification of the RBM24 3′UTR for cloning	1756
RBM24-3′UTR-R	GAATTCGAAGTTTTTAAAAATTATATTTAATAC		
RBM24-3′UTR-T7-F	GGATCCTAATACGACTCACTATAGGACCAGCCATCTGATCAAAGTTG	PCR amplification of the RBM24 3′UTR sequence for transcription in vitro	1756
RBM24-3′UTR-T7-R	GAATTCGAAGTTTTTAAAAATTATATTTAATAC		

### Biotin-labeled RNA synthesis and pull-down

Biotin-labeled RNA was synthesized via in vitro transcription as previously described^3^. The RBM24 3′ UTR containing T7 promoter sequences was amplified with or without Biotin-16-UTP (Ambion, AM8452) by using the MEGAscript T7 Transcription Kit (Ambion, AM1334). The miRNeasy Mini Kit (217004; Qiagen) was used to purify the transcribed RNA, and biotin pull-down was carried out to detect interactions between the RBM24 3′ UTR and microRNAs as previously described^19^. In brief, cells were transfected with 4 μg of biotin-labeled RNA for 24 h. Then, the cells were cross-linked with 1% formaldehyde in PBS and quenched with 0.125 M glycine. The cells were resuspended in lysis buffer on ice for 10 min and sonicated. The cell lysate was diluted two times with hybridization buffer. Streptavidin Dynabeads (Life Technologies) were blocked for 2 h at 4 °C in lysis buffer containing 1 mg/ml yeast tRNA and 1 mg/ml BSA and washed twice with 1 ml lysis buffer. Then, 100 μl of the washed/blocked Dynabeads was added to the lysate, and the mixture was rotated for 30 min at 37 °C. The beads were captured by magnets (Life Technologies) and washed five times. The beads were then subjected to RNA elution with buffer.

### Coimmunoprecipitation (CoIP) assay

Three micrograms of antibodies and protein A-agarose were added to 253 J cell lysates for 12 h at 4 °C. Then, protein A-agarose–antigen–antibody complexes were collected by centrifugation. After washing with IP buffer and extensive washing with lysis buffer, the immunoprecipitates were resolved by 10% SDS-PAGE, followed by western blot analysis.

### Proximity ligation assay

The proximity ligation assay (PLA) was performed as described previously^19^. Briefly, 253 J cells were seeded into six-well chamber slides and cultured for 24 h. Then, 4% paraformaldehyde was used to fix the slides. Anti-Runx1t1 and anti-TCF4 antibodies were used to stain the slides. Rabbit PLUS and Mouse MINUS Duolink In Situ Proximity Ligation Assay (PLA) kits were used to detect interactions between the two proteins following the manufacturer’s protocols. Fluorescence was detected using a laser scanning confocal microscope.

### Western blot analysis

Western blot analysis was carried out as described previously^20^. Protein was extracted from the cultured cells and frozen tissue samples with RIPA lysis buffer. Equal amounts of protein were run on SDS-PAGE and electrotransferred to polyvinylidene fluoride (PVDF) membranes (Millipore) that were then blocked with 5% milk for 2 h. Then, the membranes were incubated with primary antibodies overnight at 4 °C. The antibodies that were used were as follows: anti-RBM24 (1:500, 18178-1-AP), anti-STAT3 (1:1000, 10253-2-AP), anti-CDK4 (1:1000, 60186-1-Ig), anti-IGF1R (1:500, 20254-1-AP), anti-Runx1t1 (1:1000, 15494-1-AP), anti-EGLN1 (1:500, 66589-1-Ig), anti-TCF4 (1:500,22337-1-AP), and anti-β-actin (1:1000, sc-47778). The membranes were then incubated with the HRP-conjugated secondary antibody (1:5000, Rockland) for 1 h at room temperature. The blots were treated with ImmobiloTM Western (Millipore) and detected by ECL (enhanced chemiluminescence) Fuazon Fx (Vilber Lourmat). Images were captured and processed by FusionCapt Advance Fx5 software (Vilber Lourmat). All experiments were replicated three times.

### Vector construction and luciferase reporter assay

The 3′ untranslated region (UTR) sequence of RBM24 containing the wild-type form of the miR-625-5p target site was inserted into the Xho1- and EcoR1-digested psiCHECK vector (Promega Corp.). The 2-kb miR-625 promoter sequence was obtained by PCR with primers and inserted into the Mlu1- and Xho1-digested pGL3-basic vector (Promega Corp., Madison, WI, USA). A luciferase assay was performed as described previously ^21^. In brief, 293 A cells were seeded into a 24-well plate and cotransfected with the RBM24 reporter construct (wild-type or mutant) or the empty reporter vector and either miR-625-5p mimic and pRL-TK or mimic control and pRL-TK; additionally, 293 A cells were cotransfected with the pGL3-miR-luc vector or oeRBM24 or shTCF4 for 24 h. Luciferase activity was measured by the Dual–Glo Luciferase Assay System (Promega, Madison, WI) with a Flash and Glow (LB955, Berthold Technologies) reader. The specific target activity was expressed as the relative ratio of firefly luciferase activity to Renilla luciferase activity.

### Xenograft animal model

The xenograft model was generated as described previously^3,20,22^. Male BALB/c nude mice at 4–6 weeks of age (18–22 g) were purchased from Vital River Laboratory Animal Technology Co., Ltd. (Beijing, China). A total of 5 × 10^6^ stable shRBM24/shRunx1t1-infected 253 J cells were harvested via trypsinization and resuspended in 0.2 mL PBS mixed with 50% Matrigel (Collaborative Research Inc., Bedford, MA, USA); this suspension was injected subcutaneously into the right dorsal flank. The length and width of mouse tumors were measured twice a week with a calipers. Then, we used the following formula to calculate the tumor volume: tumor volume = (length × width^2^)/2. At the end of this experiment, the mice were euthanized by carbon dioxide asphyxiation. All animal experiments were approved by the Institutional Animal Care and Use Committee of Hebei Medical University (Approval ID: HebMU 20080026) and were designed to minimize suffering.

### Morphometry and histology

Human bladder cancer and normal bladder tissues were fixed with formalin solution and then processed for routine embedding in paraffin. Ten consecutive 5-μm-thick sections were prepared for hematoxylin and eosin staining. Cross-section images were acquired using a Leica microscope (Leica DM6000B, Switzerland) and digitized with LAS V.4.4 (Leica).

### Immunofluorescence staining

Five-micrometer paraffin-embedded cross-sections of tissues were subjected to immunofluorescence staining as described previously^22^. Sections were deparaffinized with xylene, rehydrated, and preincubated with 10% normal goat serum (710027, KPL, USA) followed by incubation with the following primary antibodies: anti-RBM24 (18178-1-AP) and anti-Runx1t1 (67086-1-Ig). The sections were subsequently treated with the following secondary antibodies: fluorescein-labeled anti-rabbit IgG (021516; KPL, USA) and rhodamine-labeled anti-mouse IgG (031806; KPL). In each experiment, DAPI (157574; MB Biomedical) was used for nuclear counterstaining. Images were captured using a confocal microscope (DM6000 CFS; Leica) and processed using LAS AF software.

### MTT assay

Cell viability was detected by MTT [3-(4,5-dimethylthiazol-2-yl)-2,5-diphenyltetrazolium bromide] colorimetric assay. Briefly, 253 J and J82 cells were plated in 96-well plates and treated with actinomycin (Act) for 0, 2, 4, and 8 h. Then, 20 μL of MTT reagent (5 mg/mL; Sigma-Aldrich) was added to each well and incubated for 3–4 h, and the absorbance was measured at 570 nm using a microplate reader (Thermo Fisher, USA).

### RNA immunoprecipitation (RIP) assays

253 J cells were transfected with pWPI or oeRBM24 for 48 h, and then cells were used to conduct RIP experiments using an RBM24 antibody (18178-1-AP) or IgG and the Dynabeads™ Protein G Immunoprecipitation Kit (10007D, Thermo Fisher) according to the manufacturer’s instructions. The RNA fraction isolated by RIP was quantified by a NanoDrop 2000 (Thermo-Fisher). cDNA was synthesized using an M-MLV First Strand Kit (Life Technologies) with random hexamer primers. The STAT3, GAPDH, and Runx1t1 RIP primers (Table 1) were used for qRT-PCR using the Platinum SYBR Green qPCR SuperMix UDG Kit (Invitrogen) and the ABI 7500 FAST system (Life Technologies).

### Chromatin immunoprecipitation (ChIP) assay

The ChIP assay was performed as described previously^3^. In brief, 253 J cells were treated with 1% formaldehyde for 10 min to cross-link proteins with DNA. The cross-linked chromatin was then prepared and sonicated to an average size of 400–600 bp. The samples were diluted 10-fold and then precleared with protein A-agarose/salmon sperm DNA for 30 min at 4 °C. The DNA fragments were immunoprecipitated overnight at 4 °C with anti-Runx1t1, anti-TCF4, or anti-IgG (as a negative control) antibody. After cross-linking reversal, TCF4 and Runx1t1 occupancy on the miR-625 promoter was examined. The results were determined by qRT-PCR. The ChIP primer sequences are summarized in Table 1.

### Target prediction

TargetScan (http://www.targetscan.org/vert_72/), the miRanda database (http://www.microrna.org/microrna/home.do) and RNA22 (http://cm.jefferson.edu/rna22/Interactive/)^23,24^ were used to identify potential microRNAs targeting the 3′ UTR of RBM24.

### Statistical analysis

Data are presented as the means ± SEM. The Student’s *t*-test was used to analyze the differences between two groups, and for multiple comparisons or repeated measurements, ANOVA or repeated ANOVA followed by Tukey’s post hoc test was used. *P* < 0.05 was considered statistically significant. Statistical analysis was performed using GraphPad Prism 7 software (GraphPad Software).

## Results

### RBM24 is upregulated in BC tissues and contributes to poor prognosis

**Fig. 1:**
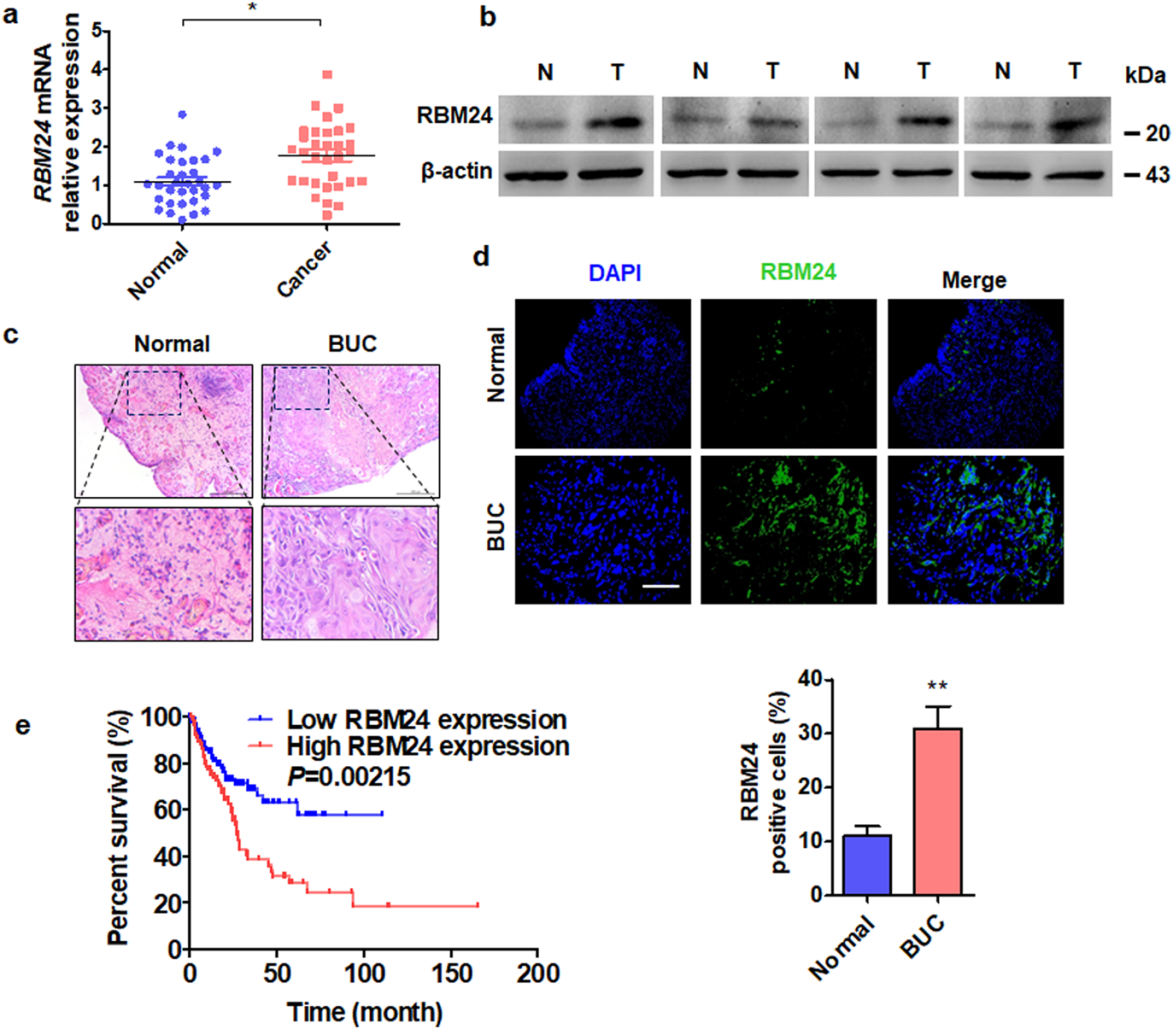
RBM24 is upregulated in BC tissues and contributes to poor prognosis. **a** RT-qPCR was used to detect the expression level of RBM24 in BC tissues (*n* = 32) and normal bladder tissues (*n* = 32). *P < 0.05 vs. normal bladder tissues. **b** Western blot analysis was used to examine the RBM24 protein level in four pairs of randomly selected tumor (*T*) and normal bladder tissues (*N*). **c** Hematoxylin and eosin staining of normal bladder and BC tissues. **d** Immunofluorescence staining of RBM24 in normal bladder and BC tissues. Scale bar = 50 μm. Bottom: statistical analysis of the percentage of RBM24-positive cells. **e** Kaplan–Meier analysis was used to analyze the overall survival of BC patients with low (*n* = 130) or high RBM24 (*n* = 130) levels from the TCGA database (cutoff value = 25%).

Our previous study revealed that RBM5 is downregulated in BC tissue^3^. In contrast, the mRNA and protein levels of RBM24 were frequently increased in BC tissue samples (*n* = 32) compared to normal bladder tissues (*n* = 32), as revealed by RT-qPCR and western blot analyses (Fig. 1a, b). Similar results were obtained by immunofluorescence staining with an RBM24-specific antibody in a cohort of 161 BC specimens (Fig. 1c, d), and correlation analysis showed that the level of RBM24 was significantly associated with tumor size and stage but not with other clinicopathologic factors, such as age, sex, or tumor grade (Table 2). Additionally, the TCGA database also revealed that higher RBM24 mRNA levels in BC patients were associated with poor overall survival (*P* = 0.00215, Fig. 1e). Together, these clinical data suggest that upregulation of RBM24 may be a critical event driving BC progression.

**Table 2:** Correlations between RBM24 mRNA expression and clinicopathological characteristics.

Characteristic	Number of patients (%)	RBM24 expression		*P*-value^a^
		Low (%)	High (%)	
**Total no. of patients**	161	89	72	
***Age (years)***				
≤63^b^	70	37 (52.86)	33 (47.14)	0.772
>63	91	46 (50.55)	45 (49.45)	
***Gender***				
Male	102	56 (54.90)	46 (45.10)	0.264
Female	59	27 (45.76)	32 (54.24)	
***Tumor size (cm)***				
≤3.0^c^	106	58 (54.72)	48 (45.28)	**0.001**
>3.0	55	15 (27.27)	40 (72.73)	
***Tumor grade***				
Low	97	45 (46.39)	52 (53.61)	0.221
High	64	36 (56.25)	28 (43.75)	
***T classification***				
Ta, T1	105	46 (43.81)	59 (56.19)	**0.041**
T2-T4	56	34 (60.71)	22 (39.29)	
***pN status***				
pN−	114	55 (48.25)	59 (51.75)	0.120
pN+	47	29 (61.70)	18 (38.30)	
***Tumor multiplicity***				
Unifocal	69	28 (40.58)	41 (59.42)	0.818
Multifocal	92	39 (42.39)	53 (57.61)	

Significant associations are shown in bold in the *p-*value column (*p-*value <0.05).

^a^Chi-square test.

^b^Median age.

^c^Median size.

### RBM24 promotes the proliferation of BC cells in vitro

**Fig. 2:**
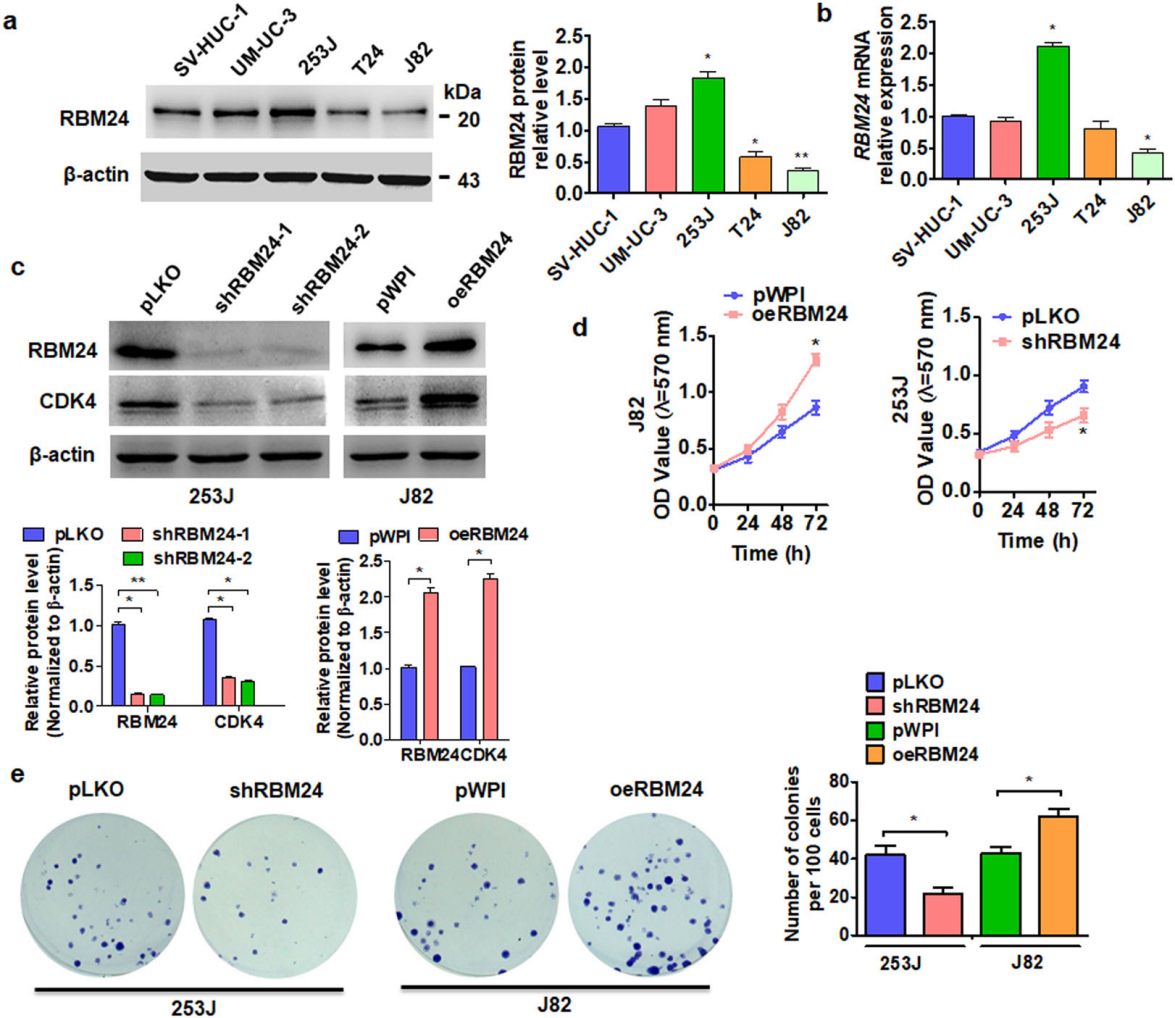
RBM24 promotes the proliferation of BC cells in vitro. **a** Western blot analysis was used to examine the protein level of RBM24 in human uroepithelial cells (SV-HUC-1) and BC cell lines (UM-UC-3, 253 J, T24, and J82). Densitometric analysis (A) was performed in three independent experiments. *P < 0.05, **P < 0.01 vs. the SV-HUC-1 group. **b** RT-qPCR was used to detect the mRNA expression of RBM24 in the cell lines. *P < 0.05 vs. the SV-HUC-1 group. **c** The RBM24 and CDK4 protein levels were measured by western blot analysis in shRBM24-transfected 253 J cells or RBM24 overexpression vector (oeRBM24)-transfected J82 cells. *P < 0.05, **P < 0.01 vs. the corresponding control. **d** Cells were prepared as in (**c**), and cell viability was measured by MTT assay. *P < 0.05 vs the corresponding control. **e** Cells were prepared as in (**c**), and cell viability was measured by colony formation assays. The right panel shows the numbers of colonies formed. *P < 0.05 vs the corresponding control.

To investigate the specific functions of RBM24 in BC, we first compared protein levels between a normal bladder cell line (SV-HUC-1) and a series of BC cell lines (UM-UC-3, 253 J, T24, and J82). RBM24 was significantly elevated in two tumor cell lines (UM-UC-3 and 253 J) but downregulated in one (J82) compared to normal bladder cells (Fig. 2a). However, the mRNA expression of RBM24 was upregulated only in the 253 J cell line and decreased in the J82 cell line (Fig. 2b). Therefore, these two cell lines were selected for subsequent loss- and gain-of-function experiments. In light of the correlation between RBM24 expression and tumor stage, we speculated that RBM24 may be involved in cell proliferation. RBM24 knockdown in 253 J cells using a specific shRNA decreased RBM24 expression compared with the shRNA control vector, while RBM24 overexpression in J82 cells via transfection with a pWPI–RBM24 vector increased RBM24 expression compared with the empty overexpression vector. In addition, RBM24 knockdown in 253 J cells suppressed the expression of the cell proliferation marker CDK4, while RBM24 overexpression in J82 cells increased CDK4 expression (Fig. 2c). Furthermore, MTT and colony formation assays revealed that overexpression of RBM24 promoted J82 cell proliferation, while RBM24 knockdown suppressed the proliferation of 253 J cells (Fig. 2d, e). Together, these data suggest that RBM24 promotes BC cell proliferation.

### Runx1t1 mediates RBM24-induced cell proliferation

**Fig. 3:**
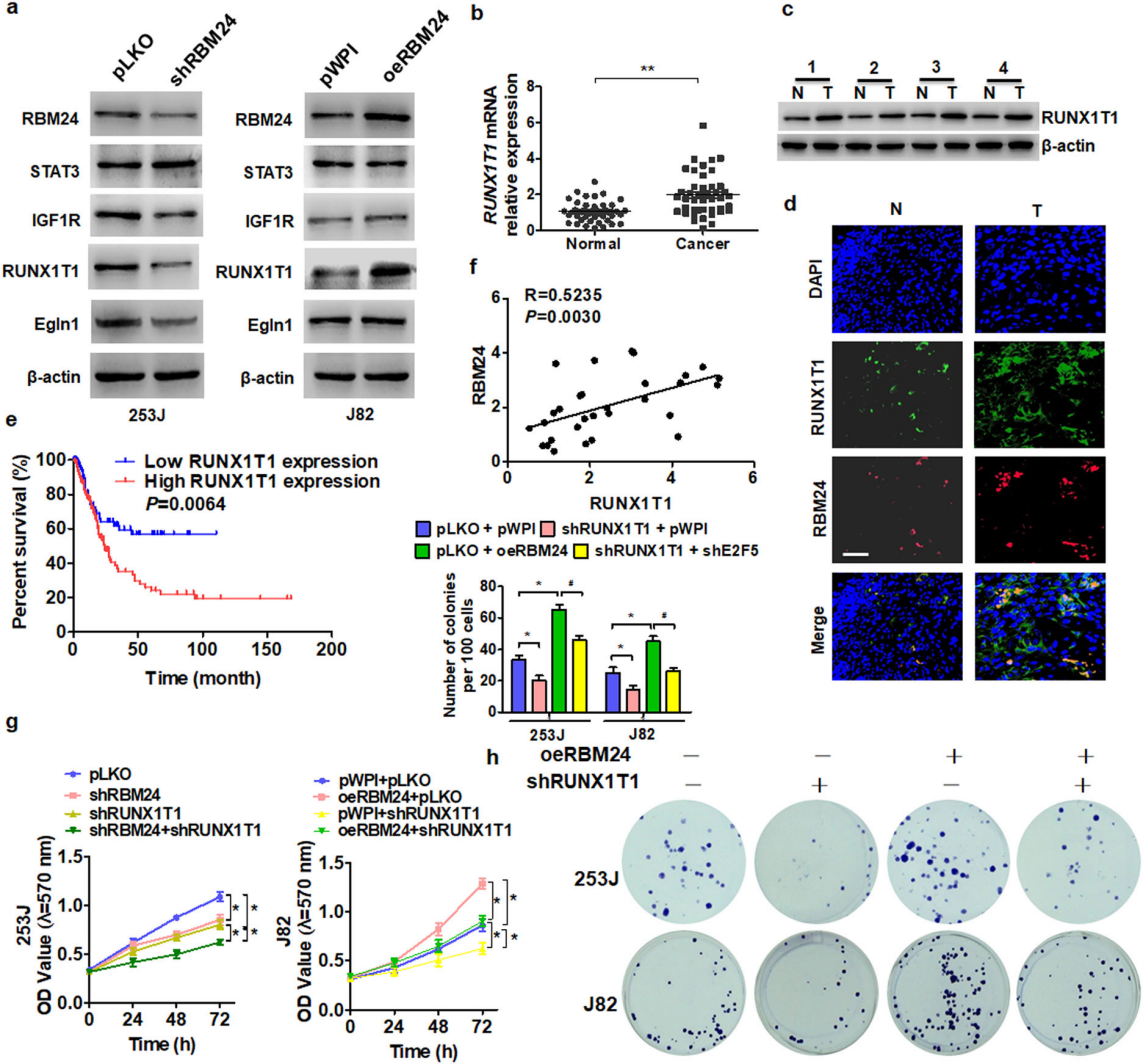
Runx1t1 mediates RBM24-induced cell proliferation. **a** 253 J cells were transfected with shRBM24 vector, or J82 cells were transfected with oeRBM24. Western blotting was used to detect protein expression with the indicated antibodies. **b** RT-qPCR was used to detect the mRNA expression of Runx1t1 in 62 pairs of BC tissues and normal bladder tissues. **P < 0.01 vs. normal bladder tissues. **c** Runx1t1 protein levels were measured by western blotting in four pairs of randomly selected tumor (*T*) and normal bladder tissues (*N*). **d** Immunofluorescence staining of RBM24 (red) and Runx1t1 (green) in the normal bladder (*N*) and BC tissue (*T*). Scale bar = 50 μm. **e** Kaplan–Meier analysis was used to analyze the overall survival of BC patients with low or high Runx1t1 levels from the TCGA database. **f** Pearson’s correlation revealed a positive correlation between the mRNA levels of RBM24 and Runx1t1 in BC tissues (*R* = 05235; *P* = 0.0030). **g** 253 J and J82 cells were transfected with the indicated vectors, and cell viability was measured by MTT assay. *P < 0.05 vs the corresponding control. **h** Cells were prepared as in (**g**), and cell viability was measured by colony formation assays. The top panel shows the numbers of colonies formed. *P < 0.05 vs the corresponding control.

Previous studies have reported that RBM24 functions as a regulator of multiple genes linked to cell proliferation, fate, differentiation, and apoptosis^10^. To investigate the molecular mechanisms underlying the regulation of BC cell proliferation by RBM24, we examined the expression levels of candidate effectors selected according to the previous studies^10^. Among the 6 candidate genes, Runx1t1 was significantly downregulated in RBM24-depleted 253 J cells and upregulated in RBM24-overexpressing J82 cells, as revealed by western blot analysis (Fig. 3a). Runx1t1 mRNA levels were significantly upregulated in BC tissue samples compared to normal bladder tissues as measured by RT-qPCR (Fig. 3b), and Runx1t1 protein levels were also significantly increased according to western blot and immunofluorescence staining (Fig. 3c, d). Moreover, human clinical data from the TCGA database of Oncolnc (http://www.oncolnc.org) revealed that higher Runx1t1 expression was associated with poor prognosis (*P* = 0.0064, Fig. 3e). Additionally, correlation analysis showed that RBM24 mRNA expression was positively correlated with Runx1t1 mRNA expression in BC tissue (Fig. 3f).

To determine whether Runx1t1 contributes to RBM24-regulated BC cell proliferation, we performed a rescue experiment. As shown in Fig. 3g, 253 J cell growth was markedly reduced after combined knockdown of RBM24 and Runx1t1 compared to that after knockdown of either gene alone. Conversely, the enhanced proliferation observed in J82 cells overexpressing RBM24 was abolished by cotransfection with shRunx1t1, as evidenced by colony formation assay (Fig. 3h). Together, these data suggest that Runx1t1 participates in the RBM24-mediated regulation of BC cell proliferation.

### RBM24 promotes Runx1t1 expression by stabilizing its mRNA

**Fig. 4:**
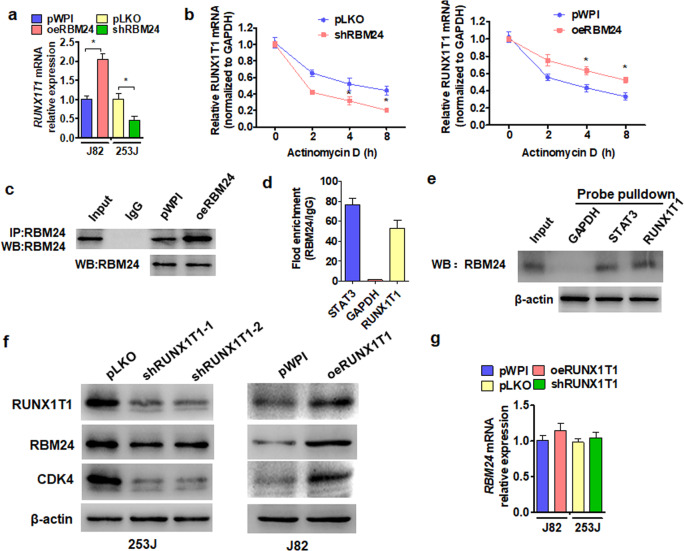
RBM24 promotes Runx1t1 expression by stabilizing its mRNA. **a** J82 cells were transfected with the RBM24 overexpression vector pWP-RBM24, or 253 J cells were transfected with shRBM24. The expression of Runx1t1 mRNA was measured by RT-qPCR. *P < 0.05 vs. the corresponding control. **b** Cells were treated as in (**a**) and then exposed to actinomycin D for 0, 2, 4, and 8 h. The mRNA expression of Runx1t1 was detected by using qRT-PCR. *P < 0.05 vs. the corresponding control. **c** 253 J cells were transfected with pWPI-RBM24 or empty vector. Western blotting was used to measure the precipitation efficiency of the RBM24 antibody. **d** RNA-binding protein immunoprecipitation (RIP) PCR was used to test the interaction between the RBM24 protein and Runx1t1 mRNA. **e** Different probes were used for oligo-pulldown, and western blot analysis was used to detect RBM24 in the pulldown precipitate. **f** 253 J cells were transfected with shRunx1t1, or J82 cells were transfected with pWP-Runx1t1. Western blot analysis was used to detect the protein levels of Runx1t1, RBM24 and CDK4. **g** Cells were prepared as in (**f**), and RT-qPCR was used to detect RBM24 mRNA expression.

Because RBM24 and Runx1t1 were positively correlated in BC, we then investigated whether RBM24 regulates Runx1t1 expression in BC cells and the underlying mechanisms. RT-qPCR results showed that overexpression of RBM24 upregulated the mRNA expression level of Runx1t1, while knockdown of RBM24 downregulated the mRNA expression level of Runx1t1 in BC cells (Fig. 4a). As previous studies have reported that RBM24 enhances RNA stability^10,25^, we investigated whether elevated RBM24 prolongs Runx1t1 mRNA expression following blockade of new transcription using actinomycin D (ActD). Indeed, RBM24 knockdown in 253 J cells reduced the duration of Runx1t1 mRNA expression, whereas RBM24 overexpression in J82 cells prolonged Runx1t1 mRNA expression, consistent with RBM24-mediated enhancement of Runx1t1 mRNA stability (Fig. 4b). To confirm this notion, we examined whether RBM24 binds to Runx1t1 mRNA using in vitro RNA pull-down and RNA-binding protein immunoprecipitation (RIP) assays. Coimmunoprecipitation confirmed that the RBM24 antibody effectively pulled down endogenous RBM24 protein (Fig. 4c), and PCR analysis of RIP products revealed the presence of the STAT3 and Runx1t1 mRNA 3′-untranslated regions (3′UTRs) but not the 3′UTR of GAPDH (Fig. 4d). Consistent with this finding, a biotinylated Runx1t1 mRNA-3′UTR and positive control STAT3-mRNA probe pulled down RBM24 protein, while a GAPDH mRNA-3′UTR probe did not (Fig. 4e). These results suggest that RBM24 can bind to the Runx1t1 mRNA 3′UTR, thereby enhancing mRNA stability.

We then used western blotting to determine whether Runx1t1 regulates the expression levels of genes associated with cell proliferation. Depletion of Runx1t1 in 253 J cells reduced the expression of the proliferation marker gene CDK4, while overexpression of Runx1t1 in J82 cells increased CDK4 protein levels (Fig. 4f). While RBM24 protein expression was positively regulated by Runx1t1 protein expression, neither depletion of Runx1t1 in 253 J cells nor overexpression of Runx1t1 in J82 cells altered RBM24 mRNA levels (Fig. 4g).

### Runx1t1 interacts with the transcription factor TCF4 to mediate RBM24 upregulation and cell proliferation

**Fig. 5:**
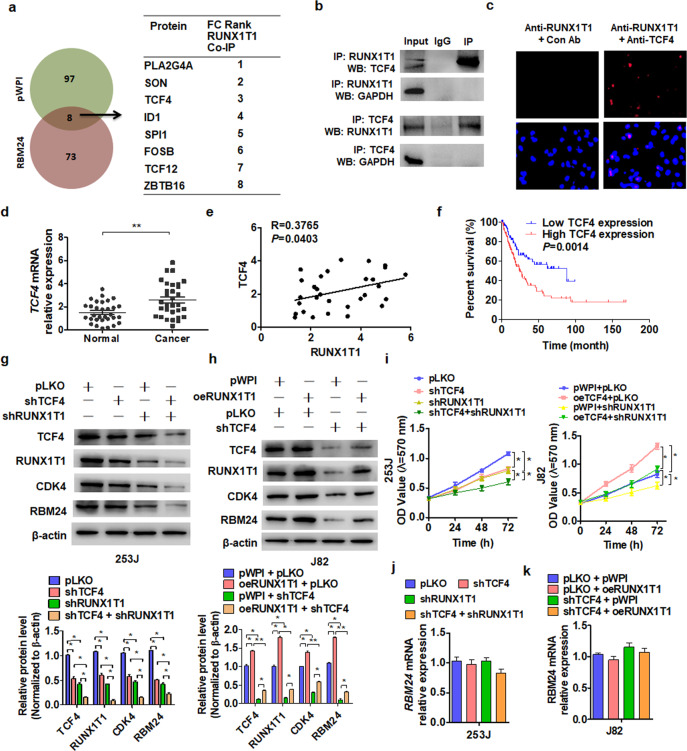
Runx1t1 interacts with the transcription factor TCF4 to mediate RBM24 upregulation and cell proliferation. **a** Coimmunoprecipitation coupled with mass spectrometry (CoIP-MS) was performed with an anti-Runx1t1 antibody in J82 cells to determine whether the proteins interacting with Runx1t1 change after RBM24 overexpression. The right table shows 8 proteins that exhibited increased interaction with Runx1t1. **b** CoIP analysis was used to detect the interaction between TCF4 and Runx1t1, and GAPDH was used as a negative control. **c** In situ proximity ligation analysis (PLA) detected the interaction between TCF4 and Runx1t1. Red color indicates PLA-positive cells. **d** RT-qPCR was used to examine the expression of TCF4 in BC tissues and normal bladder tissues. **P < 0.01 vs. normal bladder tissues. **e** Pearson’s correlation analysis of the correlation between TCF4 and Runx1t1 mRNAs in BC tissues (*R* = 0.3765; *P* = 0.0403). **f** Kaplan–Meier analysis was used to analyze the overall survival of BC patients with low or high TCF4 levels from TCGA database (*P* = 0.0014). **g**, **h** Western blot analysis was used to examine TCF4, Runx1t1, CDK4 and RBM24 expression in 253 J cells transfected with shTCF4 and shRunx1t1 either alone or together (**g**) or J82 cells transfected with oeRunx1t1 and shTCF4 either alone or together (**h**). The attac**h**ed panel shows the densitometric analysis from three independent experiments. *P < 0.05, **P < 0.01 vs. the corresponding control. **i** Cells were prepared as in (**g**, **h**), and cell viability was measured by MTT assay. *P < 0.05 vs. the corresponding control. **j**, **k** Cells were prepared as in (**g**, **h**), and RT-qPCR was used to detect the mRNA expression of RBM24.

Runx1t1 functions as a transcription cofactor by interacting with various partner proteins^26,27^. To identify the partner(s) involved in RBM24 regulation, we performed coimmunoprecipitation coupled with mass spectrometry (CoIP-MS). Eight proteins were upregulated in RBM24-overexpressing BC cells, including the transcription factor TCF4 (Fig. 5a), and reciprocal immunoprecipitation showed a strong interaction between Runx1t1 and TCF4 in BC cells (Fig. 5b). Additionally, an in situ proximity ligation assay (PLA) confirmed direct binding between Runx1t1 and TCF4 (Fig. 5c). In addition, TCF4 mRNA levels were significantly upregulated in BC tissues compared to normal bladder tissues (Fig. 5d) and positively correlated with Runx1t1 mRNA levels (Fig. 5e). Analysis of survival data from the TCGA database showed that higher expression of TCF4 was associated with poor prognosis (Fig. 5f).

To investigate whether TCF4 participates in the RBM24/Runx1t1 axis to regulate BC cell proliferation, we performed rescue experiments. Cotransfection of 253 J cells with shTCF4 and shRunx1t1 inhibited the expression of RBM24 and CDK4 to a greater extent than knockdown of Runx1t1 alone (Fig. 5g). Transfection of J82 cells with shTCF4 also sharply reduced RBM24 and CDK4 expression levels, and this reduction was reversed by cotransfection with the Runx1t1 overexpression vector (Fig. 5h). Knockdown of TCF4 and Runx1t1 also decreased the proliferation rate of 253 J cells to a greater extent than knockdown of either protein alone. Conversely, overexpression of Runx1t1 promoted J82 cell growth, an effect that was abolished by shTCF4 cotransfection (Fig. 5i). Since Runx1t1 alone did not regulate RBM24 transcription (Fig. 4g), we examined whether TCF4 regulates RBM24 transcription. However, neither Runx1t1 nor TCF4 regulated RBM24 mRNA expression or modulated the stability of the RBM24 protein (Fig. 5j, k, Supplementary Fig. 1). Together, these data suggest that Runx1t1 interacts with TCF4 to promote RBM24 protein expression rather than gene transcription.

### miR-625-5p mediates Runx1t1/TCF4-regulated proliferation by direct targeting in BC cells

**Fig. 6:**
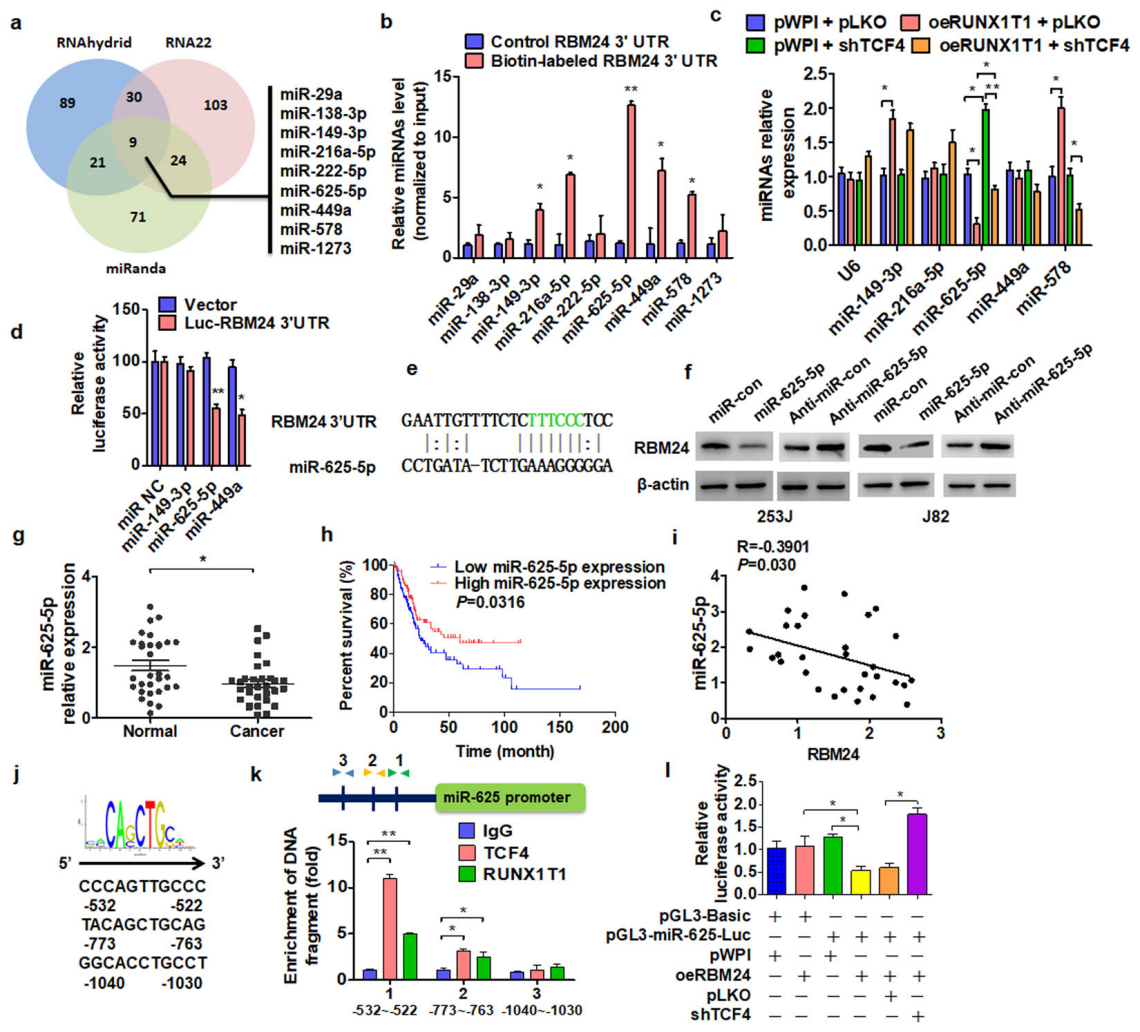
miR-625-5p mediates Runx1t1/TCF4-regulated proliferation by direct targeting in BC cells. **a** Venn diagram displaying potential microRNAs associated with the RBM24 3′UTR sequence from three online target-prediction programs. **b** Biotin-labeled RBM24 3′UTR RNA was transfected into J82 cells, followed by a biotin pull-down assay using Streptavidin-coupled Dynabeads. The miRNAs were extracted from the sedimented beads, and the relative levels of 9 candidate miRNAs were detected by RT-qPCR. *P < 0.05, **P < 0.01 vs. the control probe. **c** J82 cells were transfected with oeRunx1t1 or shTCF4 or cotransfected with both vectors together. RT-qPCR was used to detect the expression of 5 miRNAs. *P < 0.05, **P < 0.01 vs. the corresponding controls. **d** J82 cells were cotransfected with the RBM24 3′UTR and the indicated miRNA mimics. Luciferase reporter assays showed that miR-625-5p and miR-449a reduced RBM24 3′UTR luciferase activity. *P < 0.05, **P < 0.01 vs. the control vector. **e** The predicted miR-625-5p binding site in the RBM24 3′UTR. **f** 253 J and J82 cells were transfected with the indicated miRNAs, and then western blotting was used to detect RBM24 protein expression. **g** RT-qPCR was used to detect the expression of miR-625-5p in BC tissues or normal bladder tissues. *P < 0.05 vs. normal bladder tissues. **h** Kaplan–Meier analysis of data from the TCGA database was used to analyze the correlation between the overall survival of BC patients with miR-625-5p levels (*P* = 0.0316). **i** Pearson’s correlation analysis of the correlation between RBM24 and miR-625-5p expression in BC tissues (*R* = −0.3901; *P* = 0.0030). **j** Potential binding site of TCF4 in the miR-625 promoter. **k** ChIP-qPCR was used to detect TCF4 and Runx1t1 complex binding to the miR-625 promoter region in 293 A cells. *P < 0.05, **P < 0.01 vs. IgG. **l** The miR-625 promoter-luciferase reporter was cotransfected with the RBM24 overexpression vector or with the shTCF4 vector into 293 A cells. Luciferase reporter assays were performed to determine luciferase activity. *P < 0.05 vs. the corresponding control.

Our finding that Runx1t1/TCF4 regulates RBM24 protein expression without influencing RBM24 mRNA levels suggests that Runx1t1/TCF4 regulates RBM24 at the posttranscriptional level. MicroRNAs (miRNAs) are critical regulators of gene expression at the posttranscriptional level, so we conducted bioinformatics analyses using TargetScan, miRanda, and RNA22 to predict miRNA sequences targeting the RBM24 3′UTR and then used a T7 RNA transcriptase to generate an RBM24 3′UTR containing biotin-labeled uracil (Fig. 6a). After transfection, complementary miRNAs were extracted by pull-down assay, and the expression levels of 9 candidates were compared by RT-PCR. The results showed that miR-149-3p, miR-216a-5p, miR-625-5p, miR-449a, and miR-578 were enriched by the RBM24 3′UTR pull-down assay (Fig. 6b). Next, we assessed miRNA expression levels after up- and downregulation of Runx1t1 and TCF4 expression to identify the miRNAs involved in Runx1t1/TCF4-mediated regulation of RBM24 expression. As shown in Fig. 6c, only miR-625-5p was regulated by both Runx1t1 and TCF4. Furthermore, a luciferase reporter assay showed that miR-625-5p directly targeted the RBM24 3′UTR (Fig. 6d, e), and western blot analysis confirmed that RBM24 protein expression was reduced by miR-625-5p mimic transfection into BC cells compared to transfection with its antagomir (Fig. 6f). Notably, the expression of miR-625-5p was downregulated in BC tissue compared to normal bladder tissue (Fig. 6g). Moreover, analysis of survival data from the TCGA database revealed that lower expression of miR-625-5p was associated with poor prognosis (Fig. 6h). Additionally, miR-625-5p expression was negatively correlated with RBM24 mRNA expression in BC tissue (Fig. 6i).

To investigate whether the Runx1t1/TCF4 complex regulates RBM24 expression by directly suppressing miR-625-5p transcription, we first predicted the potential TCF element within the 2-kb 5′-promoter region of miR-625-5p using the Ensembl and PROMO 3.0 websites, which identified three potential TCF elements (Fig. 6j). ChIP analysis confirmed that Runx1t1/TCF4 bound predominantly to the region located –522 to –532 bp upstream of the transcription start site within the miR-625-5p promoter (Fig. 6k), and a luciferase assay yielded similar results (Fig. 6l). Together, these findings indicate that Runx1t1/TCF4 directly inhibits the transcription of miR-625-5p, thereby disinhibiting RBM24 expression.

### Disruption of the RBM24/Runx1t1/TCF4/miR-625-5p axis inhibits BC xenograft growth in vivo

**Fig. 7:**
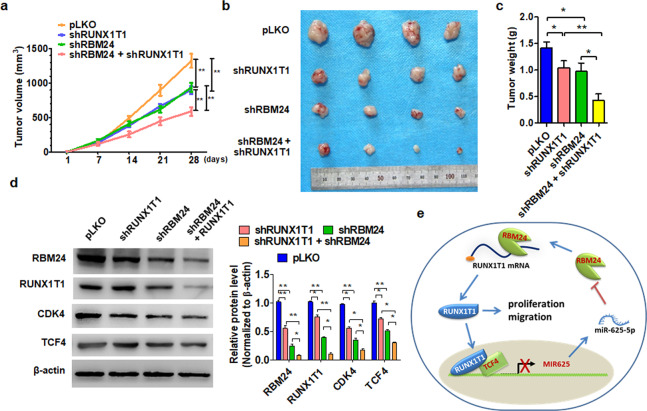
Disruption of the RBM24/Runx1t1/TCF4/miR-625-5p axis inhibits BC xenograft growth in vivo. **a** 253 J cells with stable depletion of RBM24, Runx1t1, or both together were injected subcutaneously into nude mice to establish BC xenograft tumors. Tumor volumes were monitored by direct measurement. *P < 0.05, **P < 0.01 vs. the corresponding control. **b** Representative tumor sizes in each group of mice. **c** Xenograft tumor wet weight in each group of mice. *P < 0.05, **P < 0.01 vs. the corresponding control. **d** Western blot analysis was used to measure the protein levels of RBM24, Runx1t1, CDK4, and TCF4 in xenograft tumors.

Finally, we examined whether RBM24 and Runx1t1 regulate BC cell growth in vivo using a xenograft model. Injection of 253 J cells with stable knockdown of RBM24 and Runx1t1 yielded smaller tumors in nude mice than the injection of sham-transfected 253 J cells. Furthermore, the tumor volume was much smaller in mice implanted with RBM24/Runx11 double knockdown cells than in mice implanted with single knockdown cells (Fig. 7a, b). These findings were mirrored by the mean tumor wet weights following excision (Fig. 7c). Western blot analysis of extracted tumor tissue also demonstrated that tumors derived from cells with silencing of either Runx1t1 or RBM24 exhibited significantly downregulated Runx1t1, RBM24, and CDK4 levels compared to tumors derived from control cells, and this downregulation was further enhanced by the simultaneous knockdown of both Runx1t1 and RBM24 (Fig. 7d). These findings suggest that the RBM24/Runx1t1/TCF4/miR-625-5p axis inhibits the proliferation of BC cells in vivo.

## Discussion

In this study, we identified an RBM24/Runx1t1/TCF4/miR-625-5p feedback loop that drives BC progression. First, RBM24 expression was significantly higher in BC tissues than in corresponding normal tissues, and elevated RBM24 expression was correlated with poor prognosis. Second, RBM24 overexpression promoted BC cell proliferation in vivo and in vitro and enhanced Runx1t1 protein expression by increasing Runx1t1 mRNA stability. Third, Runx1t1 interacted with the transcription factor TCF4, and this Runx1t1–TCF4 complex positively regulated RBM24 protein expression by suppressing the expression of the RBM24 negative regulator miR-625-5p, resulting in the formation of a positive feedback loop driving elevated RBM24 expression and BC cell proliferation. These findings suggest that the RBM24/Runx1t1/TCF4/miR-625-5p axis is a critical promoter of BC initiation and progression (Fig. 7e).

RBPs influence the structure and interactions of target RNAs, thereby influencing RNA biogenesis, stability, function, transport, and subcellular localization^28^. Many RBPs are expressed in a tissue-specific manner to drive developmental processes^29^. For instance, RBM24 is highly expressed in heart and muscle tissues^7^ and regulates cardiac embryonic stem cell differentiation via a splicing-mediated mechanism^30^. In addition, RBM24 was reported to suppress nasopharyngeal cancer progression^9^. In the current study, however, RBM24 accelerated BC cell proliferation both in vivo and in vitro. This discrepancy may be explained by differences in upstream and downstream regulatory factors^31^. Consistent with this accelerated proliferation, high levels of RBM24 promoted the expression of Runx1t1 and correlated with poor prognosis in BC patients.

Transcription factor 4 (TCF4) is a member of the helix–loop–helix (bHLH) transcription factor family that recognizes and binds the Ephrussi box (E-Box) DNA element (5′-ACANNTGT-3′)^32^. TCF4 regulates chromatin remodeling and transcription by recruiting histone acetyltransferases (HATs), such as p300^21^. Numerous studies have also implicated TCF4 in cancer progression. Jagrut et al. reported that TCF4 signaling was upregulated in colon cancer stem cells and promoted growth and self-renewal^33^. The expression of TCF4 is associated with breast cancer chemoresistance^34^. Additionally, high expression of TCF4 was an independent adverse prognostic factor in acute myeloid leukemia^35^. However, the expression and function of TCF4 in BC are largely unknown. In the present study, we found that TCF4 was upregulated in BC tissues and that high TCF4 expression was predictive of a poor prognosis. We demonstrated that interaction with Runx1t1 and subsequent downregulation of miR-625-5p, resulting in RBM24 upregulation, is one mechanism underlying the oncogenic effect of TCF4.

## Conclusion

The present study demonstrates that upregulation of RBM24 enhances BC cell proliferation and leads to poor BC prognosis by initiating a Runx1t1/TCF4/miR-625-5p feedback loop. These findings highlight the RBM24/Runx1t1/TCF4/miR-625-5p axis as a potential therapeutic target for BC treatment.

## Supplementary information

supplementary figure 1
