# Supplementary material for: RBM24 exacerbates bladder cancer progression by forming a Runx1t1/TCF4/miR-625-5p feedback loop
Source: Exp Mol Med. 2021 May 21;53(5):933–46. doi: 10.1038/s12276-021-00623-w (PMC8178337; doi:10.1038/s12276-021-00623-w)
Supplement: Supplementary file 1 — supplementary figure 1 [file 12276_2021_623_MOESM1_ESM.pdf]

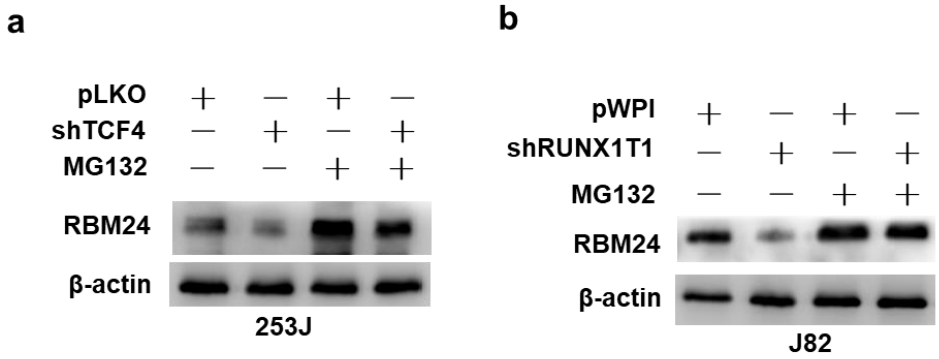

Supplementary Figure 1 **a and b** 253J and J82 cells transfected with shTCF4 **a** or shRUNX1T1 **b** or their control vector and then treated with MG132 for 24 h. Western blot detected the expression of RBM24.
